# Employers’ Perspectives of Caregiver-Friendly Workplace Policies for Caregiver-Employees Caring for Older Adults in Hong Kong: Thematic Analysis

**DOI:** 10.2196/68061

**Published:** 2025-03-31

**Authors:** Maggie Man-Sin Lee, Eng-Kiong Yeoh, Eliza Lai-Yi Wong

**Affiliations:** 1 JC School of Public Health and Primary Care Faculty of Medicine The Chinese University of Hong Kong Hong Kong China (Hong Kong); 2 Centre for Health Systems & Policy Research JC School of Public Health and Primary Care, Faculty of Medicine The Chinese University of Hong Kong Hong Kong China (Hong Kong)

**Keywords:** caregiver, aging, burnout, stress, mental health, employees

## Abstract

**Background:**

Caregiver-friendly workplace policies (CFWPs) are rare in Hong Kong. With Hong Kong facing a “silver tsunami” in the near future, it is important to understand the need for such policies and the views of employers for future facilitation.

**Objective:**

This study aimed to identify the support that is currently provided or that could be provided to caregiver-employees (CEs) caring for older adults in Hong Kong and assess the challenge and facilitative support for employers to adopt CFWPs in the specific context of Hong Kong.

**Methods:**

A qualitative research design with semistructured individual in-depth interviews with employers from Hong Kong was adopted for this study. A purposive snowball sampling method was used to recruit participants from the 7 primary industries mentioned in the Hong Kong census and from all 3 employer types (private, public, and nongovernmental organizations), which allowed the inclusion of participants sensitized to the idea and potential of CFWPs. Thematic framework analysis was used to evaluate the data collected during the interviews.

**Results:**

We interviewed 17 employers and managers from 7 major industries in Hong Kong (2.5 to 120,000 employees). There were 4 (24%) male and 13 (76%) female participants, and the participant age ranged from 30 to 50 years. All participants held managerial positions at the time of the interview. Of the 17 participants, 13 were from private companies, 2 were from public institutions, and 2 were from nongovernmental organizations. Four of the companies had a global presence. Four main themes were identified: (1) current support and potential support for CEs (which was limited to discretionary annual leave and unpaid leave when annual leave was exhausted), (2) challenges in adopting CFWPs, (3) facilitating support for adopting CFWPs, and (4) incentives for adopting CFWPs. The participants rated information and resources for CEs (mean 8.56, SD 0.37), bereavement leave (mean 8.47, SD 0.63), flexible working hours (mean 8.32, SD 0.48), and caregiver-inclusive corporate culture (mean 8.32, SD 0.48) as essential CFWPs for CEs in Hong Kong.

**Conclusions:**

While several studies have reported the types of CFWPs and their impacts on CEs, stakeholders’ perspectives on CFWPs have been rarely investigated. This study found that although employers consider CFWPs as necessary and see them as a catalyst for a long-term win-win situation, the current support for CEs is discretionary and industry-specific. Government leadership is critical for formulating, piloting, and implementing CFWPs to create a friendly environment that encourages disclosure with trust and respect across industrial sectors in Hong Kong. This study identified the current unmet needs and demands of CEs from the employer’s perspective, the barriers to large-scale adoption of CFWPs, and the path forward to inform further discourse and policy formulation in Hong Kong.

## Introduction

Filial piety has strong cultural roots in Confucian ideology and is especially prominent in Eastern countries in contrast to children-centered societies, which are more prevalent in Western countries [1-3]. However, there is some evidence of the reduction of the influence of classical Confucian ethos in Asian societies, especially in highly urbanized cities like Beijing [4,5], although the extent of this change is currently unknown. Similarly, the cultural reconfiguration of familial values and expectations has been noted among immigrant Asian families in Western countries [6].

Moreover, emerging research indicates that authoritarian filial piety, which prioritizes social obligations toward parents over one’s own needs, varies, but reciprocal filial piety, characterized by genuine gratitude for parents’ efforts or sacrifices and voluntary support for them, remains consistent across Eastern and Western cultures [3,7,8]. For instance, a study comparing filial piety among Asian Singaporean and non-Asian Australian adults showed a significantly higher mean score for authoritarian filial piety (mean 21.6, SD 5.9 vs mean 19.4, SD 6.0) on the standardized dual filial piety scale among Singaporean adults, but reciprocal filial piety (mean 34.4, SD 4.8 vs mean 34.2, SD 4.4) was comparable in the 2 populations [8].

Important differences have also been noted in patterns of filial piety within Asian cultures. For example, young adults in Taiwan endorsed strong beliefs in reciprocal and authoritarian filial piety, with both beliefs positively impacting life satisfaction, while young adults in Macau endorsed strong beliefs in reciprocal filial piety and those in Hong Kong endorsed strong beliefs in authoritarian filial piety, with positive impacts on life satisfaction [9]. Interestingly, collectivism and not ethnicity has been identified as a predictor of both forms of filial piety and positive attitudes toward caring for aging parents [7]. Hence, filial piety may be more cross-cultural than previously believed, albeit with regional nuances [7,8]. Indeed, 74% of older adults in the United States National Health and Aging Trends Study (NHATS 2011-2017) reported receiving support from one child caregiver, and 41% of children provided care to aging parents [10].

The rapid increase in the aging population has also increased the caregiving burden of family caregivers, with both being reported across cultures and countries [11,12]. Although several studies have identified caregiver burden as a significant risk factor for burnout and poor mental health outcomes among family caregivers [11,13,14], stronger filial piety may lead to lower caregiver burden [15,16]. A systematic review of 12 studies showed a significant negative correlation (*r*=–0.23) and association (β=–0.27) between filial piety and caregiver burden among adult children [15]. However, although filial piety is a prominent determinant of caregiver burden, it is not the sole determinant even in Asian societies [17].

While noting mild-to-moderate burden among Asian caregivers of older adults with chronic illnesses, a systematic review observed that specific characteristics of the care recipients (functional dependency, comorbidities, memory, and sleep impairments) and caregivers (advancing age, male gender, spouse as a care recipient, longer care duration, and lack of support/assistance) were associated with higher caregiver burden [16]. However, Asia has a lower old age dependency ratio (population aged ≥65 years to working age population) and health-adjusted dependency ratio (population with the same or higher disease burden to population with a lower disease burden as an average global 65-year-old person) than Europe, North America, or Oceania [18].

East Asia, in particular, has the highest employment-to-population ratio globally (63; for comparison, the corresponding numbers for the Euro area and North America are 58 and 60, respectively) [19], and thus, a substantially large proportion of caregivers in this region likely have a dual role of carer-employee (CE). Studies have shown that those with elderly or child caregiving responsibilities are less favorably viewed by employers in terms of competency (*F*_2_=10.99; *P*<.001; η^2^p=0.092), agency (*F*_2_=15.00; *P*<.001; η^2^p=0.121), commitment (*F*_2_=164.83; *P*<.001; η^2^p=0.602), and availability (*F*_2_=69.01; *P*<.001; η^2^p=0.388) [20]. Hence, CEs were less likely to be hired (*F*_2_=13.65; *P*<.001; η^2^p=0.111) and, if hired, were offered lower salaries (*F*_2_=13.08; *P*<.001; η^2^p=0.107) than nonprimary caregivers [20]. Moreover, the dual role adds extra strain, negatively impacting the mental and physical health of CEs [21,22] and their performance in both roles, which aligns with the role accumulation theory [14,23].

Therefore, the strain of the dual role of CEs will likely offset the low caregiver burden associated with filial piety in Asian populations [8,16,17]. Moreover, the low dependency ratio among Asian populations is likely to be mitigated by the rapid aging of many East Asian populations by 2030, as shown by the China Health and Retirement Longitudinal Study [24]. Thus, several studies have investigated the impact of caregiver-friendly workplace policies (CFWPs) on CEs, which have been associated with the improved overall health of CEs by reducing occupational and overall stress, minimizing work interruptions, and improving performance [22,25-29]. There are also direct economic benefits accruing from adopting CFWPs. For example, educating CEs about their caregiving activities generates a net benefit ranging from US $48,010 to US $675,657 for CEs and employers [30].

CFWPs that help employees manage multiple roles and improve their work-life balance [31] are increasingly being adopted to mitigate some of the caregiving burden of CEs, especially in developed economies [32]. For instance, most employers in the United States and Canada have adopted measures to create a caregiver-friendly workplace environment, such as support services, flexible working hours, financial support, and paid/unpaid leave [33,34]. Similarly, the UK government has set up programs and nongovernmental organizations (NGOs) to help the private sector become more CE friendly [33]. In the specific context of Hong Kong, official caregiver support has not yet been observed, although some private companies provide generic support [35].

Moreover, while several studies have reported the types and impacts of CFWPs [29,33], there is a paucity of studies reporting stakeholders’ perspectives on CFWPs. We could identify only 1 study that explored the perspectives of stakeholders (managers working in the Canadian health care sector) on CFWPs [36]. Therefore, this study explored the views and experiences of employers regarding CFWPs for CEs caring for older adults in Hong Kong with the objectives of identifying (1) the support that is currently provided and that could be provided to CEs by employers and (2) the challenge and facilitative support for employers to adopt CFWPs in the specific context of Hong Kong.

## Methods

### Setting

In Hong Kong, authoritative filial piety emphasizes caring for elderly parents [37]. However, the shift in parental expectations from tangible to emotional support poses challenges for caregiver mental health [38]. Sole children of aging parents face intense burdens, especially in nuclear families, which comprise 64% of households in Hong Kong [39]. One-third of households have at least one elderly member [39], and only 2.3% of the elderly population is entitled to full subsidization [40]. Additionally, given the 56% increase in children with special educational needs in the last half decade [41], support for people in the “sandwich generation” who are caring for both young children and old parents becomes important [28].

The number of CEs, defined as family members (spouse, parents, or siblings) who are employed with monetary rewards and at the same time provide care to another family member [42], in Hong Kong remains undocumented, as general informal caregiver support has only recently started to pick up in Hong Kong. Nevertheless, Hong Kong’s highly engaged workforce (50.87% of the overall population) [43] faces additional challenges due to chronic diseases among older adults. Over 65% of the older population in Hong Kong have at least one chronic disease, while one-third have ≥2 chronic diseases [44].

### Design

A qualitative research design with semistructured individual in-depth interviews involving employers and company management in Hong Kong was adopted for this study. Independent interviews helped build critical rapport between the researcher and the participants, with minimized privacy issues compared to focus groups, so that respondents would be as open and honest as possible. The COREQ (Consolidated Criteria for Reporting Qualitative Research) checklist (Multimedia Appendix 1) was used as the reporting framework [45].

### Participants

Purposive sampling was used to recruit participants, which allowed the inclusion of participants sensitized to the idea and potential of CFWPs. The list of potential participants or the sampling frame included individuals who had participated in ESG (environmental, social, and governance) policy consultations, and the information was sourced from a local NGO that advocates for working caregivers. Participants were also identified through the snowballing technique via referrals during interviews. Interviews were arranged until data saturation was achieved.

**Figure 1:**
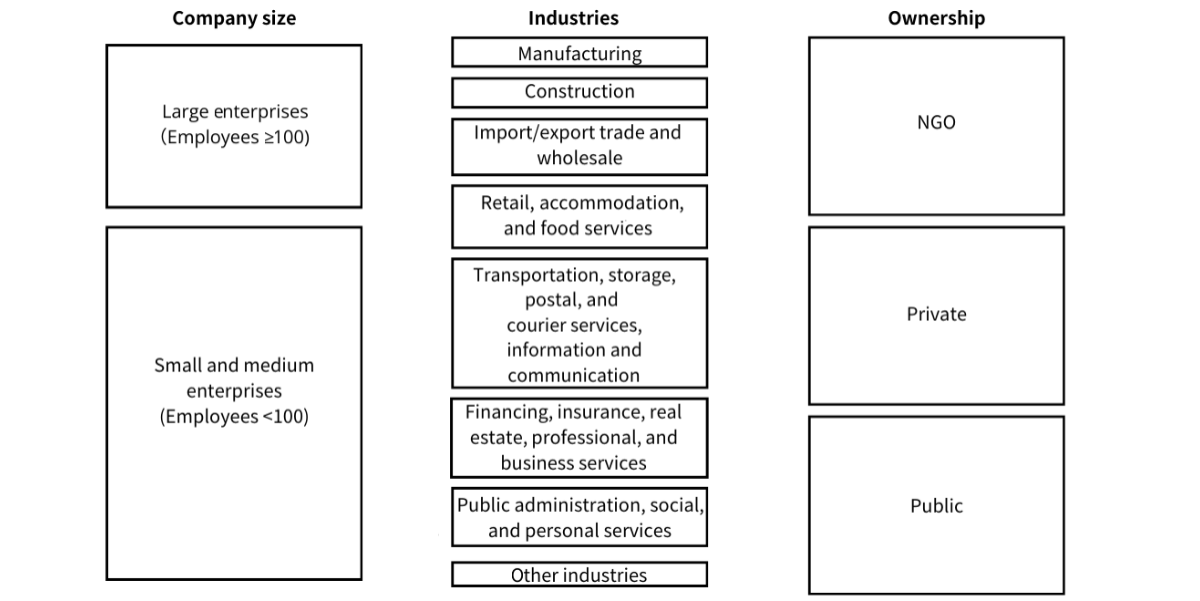
Map of employers and management recruited in this study. NGO: nongovernmental organization.

We started recruitment based on the 7 main industries in the Hong Kong census [39] and recruited from all 3 employer types (private, public, and NGO) (Figure 1). We then expanded the list to ensure a good proportion of large enterprises, small- and medium-sized enterprises, NGOs, and public institutions until data saturation was reached. Data saturation was assumed when no new themes emerged during interviews.

Participants were deemed eligible for inclusion if they had served in a managerial role for at least one staff member at a company, had experience working with CEs in a professional or personal context, and were proficient in English or Chinese (Cantonese or Mandarin). Those serving as third-party consultants for a company were excluded.

### Data Collection

The semistructured interviews were conducted using a discussion guide consisting of 17 questions related to four aspects: (1) respondents’ personal experiences with caregiver employees, (2) their attitudes and preferences for policies, (3) their perceptions of a caregiver-friendly workplace, and (4) the rating of 10 policies (Multimedia Appendix 2). The discussion guide was developed based on a thorough literature review, the first author’s previous Master’s thesis [35], and the opinions of 2 expert panels of researchers specializing in the field of caregiving (Professor E Wong, Professor D Dong, and Dr C Chan from the School of Public Health and Primary Care, The Chinese University of Hong Kong).

The first author (MMSL; PhD candidate at the Chinese University of Hong Kong) conducted all interviews in Cantonese face-to-face at the participants’ offices or through videoconferencing, with each lasting 60 to 90 minutes. Participants’ written consent and demographic data (age, gender, working position, and marital status) were obtained after the interviewer’s self-introduction (comprising qualifications, current and past research focus, and experience) and an explanation of the purpose of conducting the research. Conversation starters were used to open up the participants, so they would feel more at ease and provide the most candid answers. Prompts were also used for specific and related questions during the interview to fully explore their experiences and perceptions [46,47].

At the end of the interview, participants were invited to vote for specific CFWPs based on the degree of importance (0 to 10) from the perspective of employers and managers. The list of CFWPs developed from the global literature review included caregiver-inclusive corporate culture; paid caregiver leave; unpaid caregiver leave; bereavement leave; flexible working hours; flexible work locations; switch to part-time work; unpaid leave; medical needs/insurance of employees’ parents; and caregiving information, carer skills, and guide to community care resources. No nonparticipants were present during the interviews.

### Analysis

**Figure 2:**
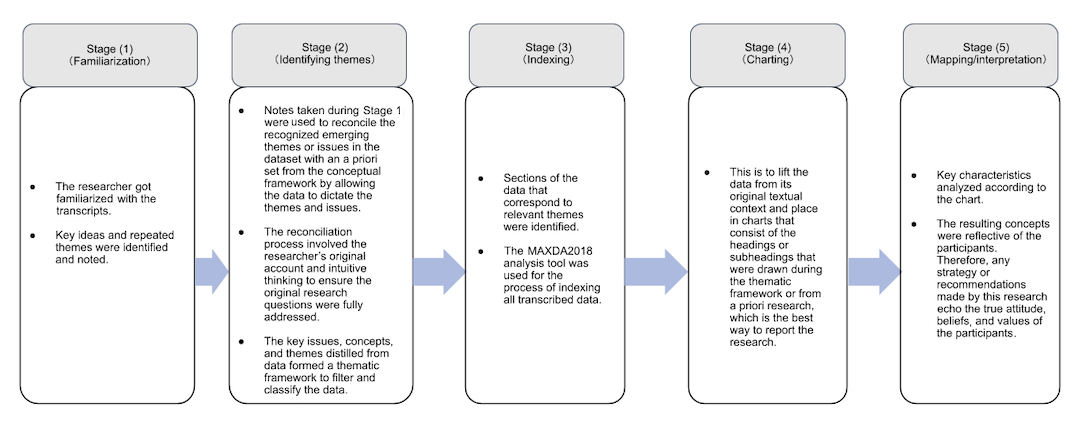
The 5-stage analytical process used in this study.

Thematic framework analysis in applied policy research was adopted for data analysis. A 5-stage analytical process described by Ritchie and Spencer [48] was used, which comprised (1) familiarization, (2) identifying a thematic framework, (3) indexing, (4) charting, and (5) mapping and interpretation (Figure 2) [48,49].

Audio recordings of all interviews were transcribed and coded using NVivo software (version 12, Lumivero) independently by 2 researchers (MMSL and EW) to ensure all interpretations were agreeable and to enhance the reliability of the study. The transcripts or the findings were not returned to participants for comment or corrections. Any discrepancy in coding was resolved by discussion with supervisors.

**Figure 3:**
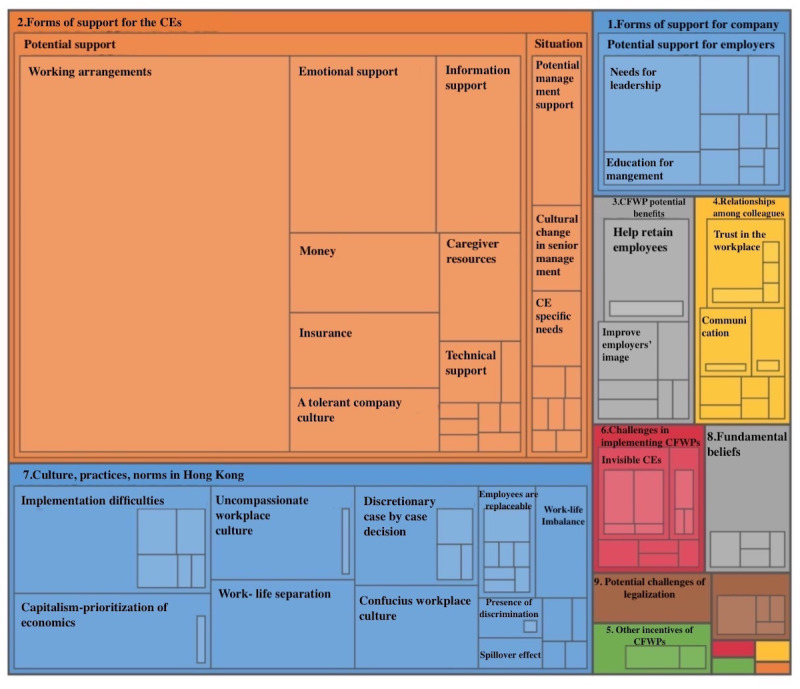
Coding hierarchy chart. CE: caregiver-employee; CFWP: caregiver-friendly workplace policy.

The rating of the 10 policies by importance was reported as mean and SD. Inapplicable policies for specific industries were removed during the calculation. For example, there were no flexible work locations for the retail, food, and construction industries. A coding hierarchy chart (Figure 3) was generated to show the visual patterns of the coding.

### Ethical Considerations

Research ethics approval was granted by The Chinese University of HK Survey and Behavioral Research Ethics Committee (reference number: SBRE(R)-21-011). Written consent was obtained from interview participants, and they were assured of their rights and freedom to withdraw from the study at any time. The information and responses of the participants were treated confidentially. All project data were anonymized and kept in password-protected folders that were only accessible to the project researchers and supervisors.

## Results

### Overview

Interviews with 17 participants comprising 4 (24%) male and 13 (76%) female participants were conducted in November 2021, and additional participants were not recruited as data saturation was achieved. Details of the participants are presented in Table 1. The age of the participants ranged from 30 to 50 years, and all held managerial positions at the time of the interview. Of the 17 participants, 13 were from private companies, 2 were from public institutions, and 2 were from NGOs. Given that the proportion of workers employed in public institutions and NGOs is less than 25% of the total workforce in Hong Kong [39], the ratio of 13:2:2 is justified.

The companies and institutions encompassed the 7 major industries in Hong Kong (ranging from 2.5 to 120,000 employees). Four of the companies had a global presence. Data saturation was achieved for NGOs and public institutions with 4 participants, all of whom viewed their industries’ organizational policies and culture similarly.

Most participants (16/17) expressed that the concepts of CFWPs had been unheard of before the interviews. However, they all supported the core ideas of CFWPs (enabling the dual roles of CEs as a caregiver and a productive member of the institution) and agreed on the fact that CFWPs were needed, subject to the specific needs of the business, for the betterment of the venture and Hong Kong’s future. The participants rated (1) information, caregiving skills, and guide to community care resources for CEs; (2) bereavement leave; (3) flexible working hours; and (4) caregiver-inclusive corporate culture as the most important policies (Table 2).

Despite the unanimity in supporting CFWPs, the operational practicality of CFWPs was of concern to all the participants. Four overarching themes were identified as outcomes of the thematic framework method in the analysis: (1) current support and potential support for CEs, (2) challenges in adopting CFWPs, (3) facilitating support for adopting CFWPs, and (4) incentives for adopting CFWPs (Table 3).

**Table 1:** Detailed demographics of the participants.

Number	Organization					Position		Age (years)/sex		Marital status^a^	
	Industry	Type	Size	Presence							
1	Public administration, social, and personal services	Public	100 (LE^b^)	Regional	Chief/director of a primary school		47/male		Married; #2		
2	Public administration, social, and personal services	Private	8-10 (SME^c^)	Regional	Executive director of a beauty company		60/female		Married; #1		
3	Transport, storage, postal, and courier services; information and communication	Private	8-10 (SME)	Regional	Director of an e-platform building firm		40-50/female		Separated; #0		
4	Import/export and wholesale	Private	8-10 (SME)	Regional	Director of an import/export company		30-40/female		Married; #1		
5	Financing, insurance, real estate, professional, and business services	Private	200-300 (LE)	Regional	Managing director of a financial company		36/female		Single; #0		
6	Public administration, social, and personal services	Private	9000 (LE)	Global	Senior marketing director of a music label corporation		48/female		Married; #2		
7	Public administration, social, and personal services	Private	2.5 (SME)	Regional	Director of an individual psychiatric clinic		40/male		Married; #1		
8	Retail, accommodation, and food services	Private	32 (SME)	Regional	Sales and marketing director		32/female		Single; #0		
9	Public administration, social, and personal services	NGO^d^	15 (SME)	Regional	Project manager		35/female		Married; #0		
10	Financing, insurance, real estate, professional, and business services	Private	20,000 (LE)	Global	District director		60/male		Married; #2		
11	Public administration, social, and personal services	Public	1300 (LE)	Regional	Head of department at a financial regulatory institution		47/female		Married; #2		
12	Public administration, social, and personal services	NGO	40 (SME)	Regional	Manager of a district health center		30-40/female		Married; #0		
13	Financing, insurance, real estate, professional, and business services	Private	100 (LE)	Regional	Senior marketing manager of a marketing company		32/female		Single; #0		
14	Public administration, social, and personal services	Private	120,000 (LE)	Global	Market access manager of a pharmaceutical company		30-40/female		Single; #0		
15	Manufacturing	Private	100 (LE)	Global	CEO^e^		30-40/male		Married; #2		
16	Construction	Private	10 (SME)	Regional	Owner of a construction company		31/female		Married; #1		
17	Retail, accommodation, and food services	Private	11 (SME)	Regional	Owner of a restaurant		31/female		Married; #0		

^a^# denotes the number of children.

^b^LE: large enterprise.

^c^SME: small and medium enterprise.

^d^NGO: nongovernmental organization.

^e^CEO: chief executive officer.

**Table 2:** Participant rating of the importance of the policies.

Rank	Policy	Score, mean (SD)
1	Information/caregiving skills/guide to community care resources	8.56 (0.37)
2	Bereavement leave	8.50 (0.55)
3	Flexible working hours	8.47 (0.63)
4	Caregiver-inclusive corporate culture	8.32 (0.48)
5	Flexible work locations	7.80 (0.61)
6	Paid caregiver leave (especially for those caring for elderly people with chronic diseases, excluding counting maternity leave)	7.72 (0.54)
7	Unpaid caregiver leave	7.60 (0.62)
8	Aiding medical needs/insurance of employees’ parents	7.56 (0.75)
9	Switch to part-time work	7.03 (0.44)
10	Unpaid leave	6.78 (0.68)

**Table 3:** Themes and subthemes.

Main theme	Subthemes
Current support and potential support for CEs^a^	Leave Nonfinancial support Emotional support Information support Technical support Industry-specific support
Challenges in adopting CFWPs^b^	Invisibility of CEs Hard to adopt a fair policy for the duration of leave Concerns about standardization
Facilitating support for adopting CFWPs	Establishment of a government compensation fund and legal framework for caregiver leave Government leadership
Incentives for adopting CFWPs	Build a positive organizational culture Recruitment of talent and extension of the worldwide network

^a^CE: carer-employee.

^b^CFWP: caregiver-friendly workplace policy.

### Theme 1: Current Support and Potential Support for CEs

Six subthemes were identified based on the participants’ current and potential support needs.

#### Leave

All participants had access to discretionary annual leave and unpaid leave when annual leave was exhausted, except participants 16 and 17, who were from the construction and food industries, which grant pay on a daily or hourly basis. All participants stated that they had the flexibility of taking a few hours of leave during working hours at their discretion for an emergency, for instance, a medical emergency related to an older family member at home. However, none of the participants reported specific caregiver policies in their workplaces.

There was bereavement leave in the participants’ companies, but as noted by participant 1, such leave “is only for immediate family members.” Participant 1 further noted the rigidity of bereavement leave as follows:

Like my wife's father, whom she also cared for, was paralyzed for a while and then passed away. I could not take leave for his funeral.

#### Nonfinancial Support

Unresolved age-related care challenges created pessimism about the future among 10 participants: rising health care costs and longer life expectancy strain lower- and middle-class families, and single-child families bear immense burdens when caring for elderly people and young people. Without the financial means to hire a helper or buy insurance policies, the quality of life of CEs and their families would drastically drop in the face of immense economic, physical, mental, and emotional strains. Employers recognized the resource-based challenges CEs face, emphasized the need for nonfinancial support, and deemed direct financial support too expensive to be on the agenda for the time being. Participant 4 specifically pointed to the importance of time as an example of nonfinancial support for working caregivers:

I think the most important thing is time. There are different degrees of illness; maybe he (i.e., the care recipient) has a stroke, or he has terminal cancer, a case like mine has only a few months left. And if you have to keep working and cannot spend time with your family. I think that's a great pity.

#### Emotional Support

Seven participants pointed to the importance of emotional support for CEs. Participants pointed to the heavy role burdens of CEs, who need to fulfill their roles as parents, team members in the workplace, and caregivers of older adults. For instance, participant 8 witnessed severe psychological distress, possibly an emotional breakdown, during one of the work trips when her colleague was struggling with poor support from her marriage and was struggling in her career while caring for her children and an ailing elderly person. Participant 4 considered that emotional support should apply to all employees (not only CEs):

Emotional support should not only apply to CEs. I think the emotions of all employees are important as long as they are in the workforce. Emotional distress is like a cold, very common.

Participant 12, an occupational therapist specializing in mental health, noted that work stress in Hong Kong is so severe that depression in young workers is often due to work stress. Long working hours and the stigma and discrimination against mental illness would further discourage Hong Kong CEs from taking care of their mental well-being. A sudden imposition of the caregiver role would further challenge the coping skills of the workers themselves and jeopardize their well-being.

#### Information Support

Four participants pointed out the need for a hotline for CEs to receive information and counseling services. Participant 14, who worked for a multinational pharmaceutical company, reported that they had already set up an international hotline to support employees in potentially all life-related aspects:

You can call there to find out about the best schools for children in your neighborhood. Since the hotline is run by a third party, anonymity is maintained to protect privacy, which makes the service even more attractive.

#### Technical Support

Four participants also pointed out the problem of time strain (high time demands and little control over timing), which applies to both work and unremitting care work. They therefore pointed to the importance of home-based care services that allow CEs some personal space and time. In this context, participant 6 mentioned that today’s open-plan offices prevent CEs from taking personal calls when needed. Therefore, personal space in the workplace, such as a phone box, would support CEs in fulfilling both roles effectively and safely. Participant 6 elaborated as follows:

Open-plan offices are very popular today, which I object to because it lacks personal space. A phone box is a personal space where colleagues have to take their own personal calls. For example, if I were to have a conversation with my mother, who is also deaf. I yell, 'No, the diarrhea pill is upstairs.' That can be very embarrassing in an open-plan office. In fact, I think this concept is in line with the idea of work-life separation.

#### Industry-Specific Support

Participant 1, one of the directors at a publicly funded primary school, pointed out that staff turnover could severely affect the quality of teaching and that CFWPs were crucial to the school context, given the overly protected privacy in the school’s workplace culture. No personal matters were allowed to be revealed that could cause suspicion and doubt among colleagues and between teachers and parents. According to participant 1, in this specific sector, either flexible working hours and locations or a switch to part-time work was possible. Given the bureaucracy in applying for leave and extended working hours, a CFWP culture for school teachers was necessary. Participant 1 commented:

Changes should be made at the top policy level of school administration. Because at the moment, even if you are a headmaster, you act according to a set of rules that restrict what you can do and what you can use.

How wages are paid is also a determinant of whether a policy of flexible working hours is needed. In construction (daily rates), insurance (piece rates), or creative industries (commission rates), the flexibility of working hours is built into the mode of payment. Therefore, there is no substantial need to include it in CFWPs. Flexible work location arrangements are not applicable in construction, retail, food, and personal services where the location of the staff is essential to the purpose of the employment.

### Theme 2: Challenges in Adopting CFWPs

#### Invisibility of CEs

Many challenges of introducing CFWPs are closely linked to the nature of CEs. A key feature of CEs is their invisibility. Unlike maternity leave, which 11 participants said is easier to implement than caregiver leave, no identifiable physical features (such as a swollen and protruding belly) make CEs recognizable. Participant 14, for instance, made the following statement:

People are very visual, maternity leave, that the colleague just had a baby. But caregivers, not everyone can see their parents or the needs of their family.

The invisibility of CEs has enormous implications for the adoption and implementation of CFWPs, as it becomes more difficult to convince everyone of the need to support CEs and make extra efforts to accommodate them, even if maternity, paternity, and caregiving leaves are all based on the same basic need to care for family members. Participant 17 elaborated as follows:

[regarding employees requiring maternity leave], we can see the belly sticking out. Also, there is a life in there; you are responsible if something happens to the mother or child. You do not feel so strong if the colleague has a sick parent, for example. If the pregnant colleague feels uncomfortable with her belly and lies down, you have to let her immediately. But if the colleague says that her father is in hospital because of stomach problems, you would not feel so strongly at that moment. Besides, I have experienced that myself—some people might doubt if it is even real.

According to the participants, CEs disclose their status as caregivers directly to their supervisors when they need to take leave or use other forms of support.

#### Hard to Adopt a Fair Policy for the Duration of Leave

The ambiguous needs of CEs create many grey areas for institutional arrangements. To be fair to all employees, the rationale for the arrangements should be clear and relevant, but this poses a problem for participants when it is necessary to allocate a certain number of leave days depending on the nature, severity, and stage of medical conditions. On the other hand, participants lacked the medical expertise to assess the CE’s situation for a case-by-case scenario, while adopting an all-caregiver-inclusive package for all CEs could be resource-intensive and disruptive to daily operations. Thus, there was no consensus among the participants regarding standardization. Participants 9 and 11, for example, felt that standardization would provide a reference point and guideline for companies, which would help combat discrimination against CEs and give the company an advantage in hiring workers. In contrast, others, such as participant 6, felt the standardization would lack the flexibility to be helpful. This complex and difficult-to-standardize situation of CEs contrasts with maternity and paternity leaves, which are standardized and predictable.

This ambiguous need of CEs further discourages companies from using CFWPs when they entail prohibitive costs in the long run. Participant 15 explained:

There is a predictable time frame for maternity and paternity leave... But sickness is a bit longer. For maternity or paternity leave, you would give birth to maybe only two to three children in this lifetime and then stop, so you would only take maternity/paternity leave two to three times; here, there is a fixed number of dates, you can predict it. But if you are taking care of chronically ill or dependent family members, it may take a long time; it may be 5 or 10 years. In between, you may have to take leave every time to accompany them to doctor's appointments, which may happen 10-20 times a year; that possibility actually exists. So, with that uncertainty, it becomes a bit more difficult to implement

the CFWP policy

#### Concerns About Standardization

Standardization also raises concerns that the mechanisms of CFWPs could be abused. Participant 16 pointed to the difficulty of proving caregiver status:

Can I just find any aunt or distant relative? That is, the moment I want to take a holiday, the moment I do not want to take this job, I can just find a random aunt. They give me a paper, I give it to the company, then I get the benefits. I feel that these grey areas can be abused very easily... If I introduce care leave as an employer, I may suddenly get them [these medical certificates]. The next day, I wake up and suddenly get a message that I can not go to work [because of a family member's condition]... The wheel would stop turning; we would not know what to do, so I think this thing is more difficult than paternity leave. With paternity leave, you can easily prove that you are the father. Due to the very ambiguous nature of CEs' needs, this can be a major trust issue for management to support CEs.

Participants commonly expressed that it was difficult to justify the CFWPs giving priority exclusively to workers caring for older individuals, risking favoritism over other needs, such as workers raising their children. The heavy workload of CEs for ailing older individuals would not make caring for needy young individuals less stressful. The cultural preference for newborns in Hong Kong was pointed out by participant 4 as follows:

Everyone expects a happy baby, but not everyone would consider the possibility of having a sick parent.

The comments make the different treatment of CEs with an ailing older relative even more challenging to accept.

### Theme 3: Facilitating Support for Adopting CFWPs

#### Governmental Compensation Fund and Legal Framework for Caregiver Leave

Addressing paid/unpaid leave for caregivers involves considering financial costs, a central issue raised by all participants. Participants proposed government compensation funds for employers to cover the costs of CEs’ time off, cashing out the mandatory provident fund (MPF) during a medical crisis, legal frameworks for eligibility, and compulsory employment insurance. Regarding the cash out from the MPF, participant 17 commented:

The original intention of the MPF was to maintain a certain quality of life in retirement. But if he (CE) can't get through the hardest time of his life (caring for ailing older family members), it doesn't matter how much money he'll receive in the future.

#### Government Leadership

The practical challenges practicing doctors face include a lack of government guidance and reimbursement. Many participants felt that the government must take the initiative to support CEs. Many participants expressed the opinion that “if the government implements it first, we will follow;” however, there is currently no single, consistent CFWP in Hong Kong. Therefore, support for CEs has only been granted on a discretionary and case-by-case basis. If enacted, the policy and legislation supporting CEs would be mandatory for them to follow, and this government initiative would even trigger a shift in companies and management thinking. Participant 2, for example, said the following:

I do not think there was anything like maternity leave before; it's even more similar with paternity, but we all have that now, so I would think, why not? [I am] also very, very supportive

of CE leave

Two participants also felt that with the introduction of maternity and paternity leaves, it would be easier to advocate for support for caregivers if the government takes the lead in implementing policies. Participant 2 commented as follows:

There is paternity leave now. Before, it was difficult, but now it is possible. If this was applied to CEs, maybe it would be quicker [to get support for them] because people have already taken a step forward.

This was also affirmed by participants 3 and 8, showing some level of readiness for CFWPs in Hong Kong, but it is crucial for the government to take the lead in getting this process started.

In the context of introducing a legal framework to support the future implementation of CFWPs, participant 11, a department head of a financial regulatory institution, highlighted the earlier success of the pilot project on maternity leave before it was legalized for all companies:

The Government could test the leave program for CE and other CFWP in some selected departments to identify the need and the possibilities of implementation. After consultations with various sectors of the economy, I believe it would be readily adopted.

All participants expressed willingness to introduce some forms of CFWPs if the government could first demonstrate the benefits of the measures and set the framework for practice. Participant 13 felt that a pilot phase within the government is a guarantee for the private sector and is one of the many conditions for local businesses to consider CFWPs.

Four participants were aware of the importance of the government communicating the needs of CFWPs to the business sector, particularly for management understanding, including recognition of the role of CEs, potential caregiver burnout, disease progression, management of older people, and stigma/discrimination against CEs. Participant 6, a director at a multinational company, emphasized that the human resources department could take the lead in advocating for CFWPs in the cultural and operational contexts to ensure sustainability and enforcement. Participants 4 and 6 recommended using case studies by the government or those in business to explore the positive changes associated with CFWPs and motivate other companies to create a caregiver-friendly workplace.

### Theme 4: Incentives for Adopting CFWPs

#### Build a Positive Organizational Culture

The improvement in loyalty and sense of belonging that CFWPs bring in the long term was acknowledged. Participant 10, a district director of a financial institution, noted the following:

supporting CEs will result in productivity losses—as an immediate loss to the company, but in the long run, the benefits of improving loyalty and sense of belonging would outweigh the costs overall.

Participants 2 and 5 noted positive spillover effects, specifically, work engagement improving the caregiving experience. CFWPs aim to facilitate a balance between the dual roles, allowing for healthier management of CEs’ circumstances, which is an incentive for adoption. Participant 5 commented:

It's better to stay at work than to stop for caregiving because you still get to see what's going on in the world, and your brain keeps moving.

Round-the-clock caregiving duties could be more stressful than taking on dual roles.

CFWPs could be seen as a confidence-building exercise between the management and employees. Management’s effort to cultivate a caregiver-friendly workplace culture is a prerequisite for gaining CEs’ trust before disclosing their status and asking for consideration for their individual needs. Participant 6 referred to her experience as follows:

When my employee came to my office and asked for a day off to take care of his parents, he must have trusted me that he was asking for help. I am very grateful for that trust.

To strengthen trust, participant 4 suggested using a medical certificate (similar to a carer’s passport scheme in the United Kingdom [50]) confirming the caregiving role of the employee, with constant updates on the care recipient’s illness, and indications of possible commitments and the durations of such commitments. Participant 3 was aware of the guilt of CEs about not being able to fulfill the dual roles simultaneously. This sense of guilt could not only hinder productivity but also encourage the suppression of emotions and interfere with communication.

#### Recruitment of Talent and Extension of the Worldwide Network

Most participants explicitly expressed their belief that CFWPs could give companies an advantage in recruiting talent. Participants considered that CFWPs allow for the retention of highly qualified staff and thus more sustainable productivity, as well as a potential reduction in training and recruitment costs. However, participant 12 stressed that the attractiveness of CFWPs might be limited among younger workers.

Participant 14 emphasized that if nonfinancial reporting is considered, CFWPs could make a company a worthwhile investment for potential investors under ESG standards:

Impact investing is now becoming more popular. Many global companies have already included ESG in their reporting; we are already lagging behind a lot, although our company has already set up a hotline for general employee concerns.

For Hong Kong, there are overall benefits of companies taking up CFWPs, namely keeping workers employable rather than out of the workforce for a significant duration, attracting foreign talent to Hong Kong, and reducing public health costs when CEs perform caregiving duties more efficiently and effectively.

## Discussion

### Principal Findings

Although support for CEs in Hong Kong is currently discretionary and depends on the context of industries, this study identified strong motivations for Hong Kong employers to adopt CFWPs. While improving mental well-being can lead to a direct improvement in productivity [51-54], the benefits of CFWPs may be more extensive than anticipated. Beyond indirect operational incentives, such as employee loyalty and talent retention, CFWPs could make a company an attractive investment for socially conscious investors under ESG standards [55]. Furthermore, CFWPs can have a positive spillover effect with a potentially significant impact on CEs’ mental well-being, especially when caregiver-inclusive corporate culture is more explicit than making decisions on a case-by-case basis.

Although this study identified several gaps and barriers to the large-scale adoption of CFWPs in Hong Kong, there is ample precedence of organizational changes in Hong Kong’s public and private sectors. For instance, the 8-step theory of change by Kotter [56], which involves the following steps: (1) create urgency, (2) build a guiding coalition, (3) form a strategic vision, (4) communicate the vision, (5) empower others to act on the vision, (6) generate quick wins, (7) sustain momentum, and (8) institutionalize new approaches, has been shown to be applicable across Hong Kong’s monolithic civil service organizations [57], publicly listed corporations [58], and NGOs [59,60], which represent the 3 primary stakeholders for the large-scale adoption of CFWPs in Hong Kong.

In line with the theory by Kotter [56], employers and management participants in this study echoed the prerequisites for the government to take the leadership in raising awareness of the needs of CEs (step 1: create urgency) and advance the discussion on CFWPs among stakeholders (step 2: build a guiding coalition) to develop (step 3: form a strategic vision) and implement (step 4: communicate the vision) goal-oriented strategies. Government leadership is indispensable for small and medium enterprises as, unlike large enterprises, they lack the in-house resources and experiences to formulate relevant policies and account for more than 98% of businesses in Hong Kong [61] (step 5: empower others).

The piloting of CFWPs in the management of public institutions seems to be an appropriate starting point, as this is where the bureaucratic burden is most remarkable (step 6: generate quick wins). CFWPs could be better thought through in this complex environment before being rolled out to the private sector and NGOs. Notably, the government has recently launched a 2-year pilot program to subsidize caregivers from low-income families caring for older individuals with moderate and severe impairments (at least 3 hours of care per day) to the tune of HK $3000 (US $386) per care recipient [62]. However, the number of households benefiting from such high thresholds is questionable, and the policy does not extend to caregivers who continue to work and contribute to the economy.

Furthermore, our participants indicated the need for a general CFWP with some specific policies for certain industries (step 7: sustain momentum). For instance, information, caregiving skills, and guide to community care resources; bereavement leave; and caregiver-inclusive corporate culture can apply to all industries. With Hong Kong well-positioned as a knowledge-based economy [63], these CFWPs may be immediately relevant for human capital and knowledge-intensive industries, such as social and personal services, professional and business services, financing, insurance, and real estate. However, flexible working hours or locations depend on industry-specific payment arrangements and the individual requirements of the working environment. For instance, construction, retail, accommodation, and food service sectors may require special considerations due to their demographics (eg, relatively younger workers) and wage payment mode (eg, piecework in construction and hourly in food services).

According to the rated importance, providing information resources, cultivating a caregiver-inclusive corporate culture, and introducing flexible working hours and locations could be piloted within the government structure to provide feedback for future public implementation (step 8: institutionalize new approaches). In addition, legalizing paid or unpaid caregiver leave is necessary. While the government reimburses companies for maternity and paternity leaves, there are other options to facilitate the future legalization of caregiver leave, such as cashing out from the MPF and introducing a legal framework on the qualifications and durations of leaves for CEs. However, cashing out from the MPF could jeopardize the original intention of securing financial security in retirement. Thus, a greater government-industry engagement focused on CFWPs is essential.

Our study underscores the need for consensus among stakeholders on caregiver eligibility criteria, including the care recipient’s age, care forms, employment conditions, relationships, and medical needs. Quantifiable parameters can ensure fairness and prevent policy misuse. For instance, the voluntary leave-sharing program in the United States allows workers to donate their unused annual leaves directly to another worker who is experiencing a personal or family medical emergency and has exhausted available paid leaves [64,65]. However, the program requires a significant income loss (at least 24 hours for full-time employees) due to illness after exhausting paid leaves [64,65]. Such a program with objective and fair parameters may be considered for Hong Kong’s different industrial sectors. Furthermore, such programs may be instrumental in breaking the status quo that personal or family matters do not belong in the workplace and may enable generous interworker benefit transfers.

There is some international precedence in identifying the pathway to develop and implement CFWPs in Hong Kong. For instance, the advocacy of CFWPs is typically supported by NGOs working with governments and legislatures to inform and influence policy [66-68]. While such advocacy groups do not exist in Hong Kong yet, they can be a vital conduit for engaging with CEs and negotiating with the government to address their most pressing needs. Similarly, Canada offers caregivers the world’s longest unpaid compassionate leave (26 weeks) [69,70]. Caregiver leave to attend to an ailing parent is covered by compulsory employment insurance, which is funded by worker premiums, without burdening businesses directly [70]. Similarly, the Japanese government has subsidized 93 caregiver leave days for the business sector [71].

Notably, the legalization of caregiver leave in Taiwan and Japan was in response to an alarming number of caregivers committing suicide or murdering elderly members under overwhelming “caring fatigue” [72]. Hong Kong should urgently consider CFWPs before caring for the needs of the aging population takes too heavy a toll on the working population. Although successful precedents in other countries can be a good starting point, it is essential to consider the unique needs and expectations of CEs and their employers in Hong Kong. For instance, while unpaid caregiver leave is legalized for CEs in Taiwan and Japan [68,71], employers in this study emphasized the importance of paid leave over unpaid leave in line with maternity and paternity leaves in Hong Kong.

### Limitations

Our study has several limitations that must be noted. First, our participants were predominantly recruited using the chain-referral method, which is known to introduce selection bias [73]. Thus, the findings may have lower generalizability, representativeness, or external validity than research conducted with a random sample [73]. However, our goal was to identify perceived barriers to CFWP implementation and galvanize further research in this domain, which may, in turn, identify additional barriers. Thus, in this type of research, the inherent risk of selection bias due to chain-referral sampling is not expected to skew the findings significantly. Second, some industries may be underrepresented in our study. For instance, while data saturation was achieved with at least two participants for each category by size (ie, small and medium enterprises and large corporations) and constitution (ie, NGOs, public institutions, and private companies), there was at least one participant from each of the main industrial sectors in Hong Kong. Third, there was no “third party” interviewer to reduce the impact of the researchers’ biases and beliefs [74] about the phenomenon (ie, CFWPs) on the quality of the data collected. Finally, this research was conducted at the peak of the COVID-19 pandemic, which could have influenced the participants’ perspectives and positions on CFWPs [75]. In addition, the pandemic conditions may have led to the unavailability of participants with diverse opinions [75].

### Conclusion

Employers and management recognize the importance of CFWPs, but current support for caregivers is discretionary and industry-specific. Government leadership is crucial for the future implementation of CFWPs, and piloting such policies within the government can drive institutional change. Ongoing consultation with the business sector on the specific needs of particular industries is essential before legalizing unpaid or paid caregiver leave in Hong Kong.

## Appendix

Multimedia Appendix 1 COREQ (Consolidated Criteria for Reporting Qualitative Research) checklist.

Multimedia Appendix 2 Discussion guide.

## Abbreviations

CE: carer-employee
CFWP: caregiver-friendly workplace policy
ESG: environmental, social, and governance
MPF: mandatory provident fund
NGO: nongovernmental organization
